# Cystitis in guinea pigs (*Cavia porcellus*): Clinical findings and treatment outcomes

**DOI:** 10.1002/vetr.70391

**Published:** 2026-02-11

**Authors:** Anika Mische, Kerstin Müller

**Affiliations:** ^1^ Small Animal Clinic Freie Universität Berlin Berlin Germany; ^2^ AniCura Veterinary Clinic Haar Haar Germany

## Abstract

**Background:**

This retrospective study examined the incidence, diagnostic methods, bacterial isolates, treatments and outcomes associated with cystitis in guinea pigs presented at a single clinic between 2009 and 2022.

**Methods:**

The clinic's medical records database was reviewed using *easyvet* software to identify cases eligible for inclusion in the study. Inclusion criteria were leukocyturia and/or bacteriuria, with at least one radiographic and one ultrasonographic examination. Culture‐confirmed cases with imaging‐based diagnoses were also included.

**Results:**

Forty‐six guinea pigs met the inclusion criteria. Clinical signs included haematuria (*n* = 12), incontinence (*n* = 10), stranguria (*n* = 5), abdominal discomfort (*n* = 6), inappetence (*n* = 8), weight loss (*n* = 4) and apathy (*n* = 3). Ultrasonography revealed ascites (28%), peritonitis (33%) and urinary bladder sludge (63%). Bacterial isolates were obtained in 21 cases, with *Corynebacterium renale* (50%) and *Staphylococcus* spp. (21%) being most frequently isolated. Resistance to fluoroquinolones was observed, while the highest susceptibility was observed with chloramphenicol. Recurrence/reinfection occurred in 37% of cases, and the median survival time was 249 days. Euthanasia owing to severe cystitis was required in 24% of cases.

**Limitations:**

The absence of urine culture in 25 cases reduced diagnostic certainty. The retrospective nature limited full assessment of antimicrobial resistance patterns.

**Conclusion:**

Cystitis involving *C. renale* was linked to chronic progression and high recurrence rates.

## INTRODUCTION

Cystitis is a common urinary tract disease in guinea pigs, primarily affecting older females.^1^, ^2^, ^3^ Urolithiasis is frequently observed as a concurrent condition.^4^, ^5^ In guinea pigs, cystitis tends to be chronic and often unresponsive to treatment,^1^, ^6^ whereas in dogs and cats, sporadic bacterial cystitis is typically uncomplicated and treatable.^7^ However, the reasons for these interspecies differences remain unclear. Possible contributing factors include the presence of urolithiasis—which may act as a nidus of infection and, through mechanical irritation and mucosal trauma, facilitate bacterial adherence and proliferation within the urinary tract^2^, ^8^—antimicrobial resistance in bacterial isolates,^4^ and the species’ stoic nature, which delays the recognition of clinical disease.^9^

In clinical practice, diagnostic and therapeutic procedures are primarily derived from those used in dogs and cats, where urinary tract infections (UTIs) are typically diagnosed using a combination of urinalysis, urine culture and imaging techniques,^10^ and are categorised as uncomplicated or complicated based on the presence of underlying abnormalities or recurrent infections.^11^, ^12^ However, such structured classifications and treatment protocols have not yet been established for guinea pigs.

This study aimed to analyse the incidence, diagnostic approaches, bacterial isolates, treatment strategies and clinical outcomes of cystitis in guinea pigs to elucidate its pathogenesis. We examined the clinical course of cystitis, distinguished between uncomplicated and complicated cases, and evaluated recurrence and reinfection frequencies. Additionally, we evaluated potential predisposing factors and comorbidities, such as glucocorticoid administration and urolithiasis, which have been reported in dogs and cats.^7^, ^13^

## MATERIALS AND METHODS

### Patient selection, data acquisition and evaluation

Medical records from AniCura Veterinary Clinic Haar were retrospectively reviewed using *easyvet* software (VetZ, 2023). The database was searched for various spellings and diagnostic terms related to UTIs in guinea pigs. Cases from 2009 to 2022 were included in the analysis.

The inclusion criteria required guinea pigs to have undergone at least one radiographic and one ultrasonographic examination, along with urinalysis indicating signs of cystitis (leukocyturia and/or bacteriuria). Additionally, animals with culture‐confirmed cystitis who had received at least one form of imaging (radiography or ultrasonography) were also included.

**FIGURE 1:**
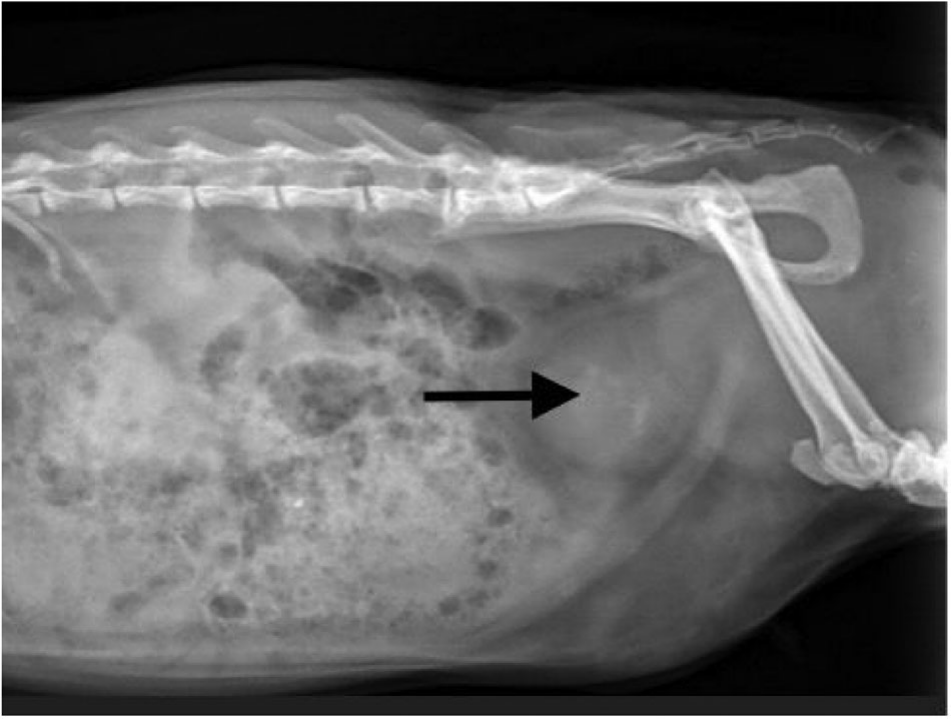
Radiograph (right lateral projection) of the caudal abdomen of a guinea pig with a mineral‐dense shadow in the centre of the urinary bladder (black arrow).

For each case, data were collected on the reason for presentation, signalment, bodyweight, comorbidities and diagnostic tests performed. The tests included general clinical examination, urinalysis, radiography, urine culture, ultrasonography, haematology and plasma biochemistry. Urinalysis findings at initial presentation were assessed based on the urine collection method (manual expression or cystocentesis), with parameters recorded as either ‘present’ or ‘unknown’ (not detected or not documented). The presence of urinary constituents was determined by cytological examination; in some instances, additional assessments were conducted using Combur‐Test urine test strips (Roche Diagnostics) and urine pH was measured using pH indicator paper (pH‐Fix, Macherey‐Nagel) (see Table 3). Radiographs were also evaluated for radiographically detectable urolithiasis (Figure 1). Treatment details (both medical and surgical), clinical outcomes (such as recurrence or reinfection rates), duration of clinical sign resolution and follow‐up period were documented. The chronological sequence of the diagnostic procedures was recorded to ensure a standardised assessment of disease progression.

All medical records were reviewed and re‐evaluated to ensure consistency in data formulation and interpretation. The dataset was compiled using a commercially available spreadsheet program (Microsoft Excel 2021).

### Statistical analyses

All analyses were performed using Statistical Package for the Social Sciences (version 31; IBM). Given the small sample size, data were analysed primarily using descriptive and nonparametric methods.

The normality of continuous variables (age and bodyweight) was assessed using the Shapiro–Wilk test. Differences between observed haematological and biochemical parameter values and predefined reference values were tested using the Wilcoxon signed‐rank test.

Survival analysis was performed using the Kaplan–Meier method, and survival distributions between surgical groups (cystotomy vs. urethrotomy) were compared using the log‐rank (Mantel–Cox) and Breslow (generalised Wilcoxon) tests. Because of the limited number of cases and events, no multivariate Cox regression modelling was conducted. All tests were two‐sided, and a *p* value of less than 0.05 was considered statistically significant.

## RESULTS

### Patient demographics

Of 133 guinea pigs suspected of having cystitis, 46 (34.6%) met the inclusion criteria. The clinical signs and lifestyle conditions of the included animals are summarised in Table 1. Female guinea pigs were over‐represented. The Shapiro–Wilk test showed that age (*p* = 0.280) and weight (*p* = 0.437) were normally distributed. The mean age was 4.7 ± 1.5 years, and the mean weight was 1.03 ± 0.15 kg. Information regarding housing type (indoor or outdoor), breeding status and frequency of litter changes or cage cleaning was insufficient in most cases. Most animals were housed in groups (59%, 27/46).

**TABLE 1:** Signalment and lifestyle conditions of 46 guinea pigs with clinical signs of cystitis.

Variable	Frequency (*n*)	Percentage (%)
Age (years)		
<3	5	10.9
3–6	34	73.9
>6	7	15.2
Sex		
Male entire	1	2.2
Male neutered	1	2.2
Female entire	38	82.6
Female spayed	6	13.0
Weight (g)		
<1000	20	43.5
>1000	24	52.2
Unknown	2	4.3
Guinea pigs per household		
Single guinea pig	2	4.3
Two guinea pigs	8	17.4
Three or more guinea pigs	27	58.7
Unknown	9	19.6

One or more clinical signs of lower urinary tract disease were observed in 42 guinea pigs (91.3%) (Table 2). The onset of clinical signs was reported by owners to occur within less than 1 week in most cases, and 16 patients were presented to the clinic on the same day that clinical signs appeared. In the remaining cases, the duration of clinical signs ranged from 1 week to 1 year, except for five instances in which owners were unable to estimate the time of onset.

**TABLE 2:** Prevalence of lower urinary tract signs in 46 guinea pigs with clinical signs of cystitis.

Clinical feature	Frequency (*n*)	Percentage (%)
Gross haematuria	16	34.8
Stranguria	5	10.9
Urinary incontinence	12	29.1
Dysuria	2	4.3
Caudal abdominal discomfort	23	50

### Diagnostic workup

#### Urinalysis

In total, 206 urine samples were collected from 46 guinea pigs, of which 203 were spontaneous specimens obtained via manual bladder expression, and three were collected by cystocentesis. Follow‐up dipstick and cytological examinations were performed in 33 guinea pigs. These results were used as controls to assess treatment effectiveness and monitor disease progression. The findings from the initial urinalysis are presented in Table 3.

**TABLE 3:** Urinalysis findings at initial presentation in 46 guinea pigs with clinical signs of cystitis, categorised by the urine collection method.

Urinalysis parameter	Manual bladder expression (*n* = 45)	Cystocentesis (*n* = 1)
Bacteria		
Cytological	18 (40%)	1 (100%)
Unknown	27 (60%)	0
Leukocytes		
Stick	2 (4.45%)	
Cytological	41 (91.1%)	1 (100%)
Unknown	2 (4.45%)	0
Erythrocytes		
Cytological	23 (51.1%)	1 (100%)
Unknown	22 (48.9%)	0
Crystals		
Cytological	22 (48.9%)	1 (100%)
Unknown	23 (51.1%)	0
Protein		
Stick	4 (8.9%)	
Unknown	41 (91.1%)	1 (100%)
pH		
Stick	5 (11.1%)	
pH 8	4 (8.9%)	
pH 9	1 (2.2%)	
Unknown	40 (88.9%)	1 (100%)

*Note*: ‘Unknown’ refers to findings that were either not detected or not documented in the medical record.

The interval between examinations varied among subjects. Crystalluria was identified in 30 of 46 animals (65.2%). Leukocytes were persistently present on urinalysis following recurrence in all guinea pigs.

#### Bacterial isolates

In total, 24 urine bacterial cultures were obtained from 21 guinea pigs (Table 4). The timing of microbiological examination varied considerably, ranging from the day of presentation to up to 1 year after the onset of clinical signs. In eight urine samples with cytological evidence of bacteria, three of which were collected via cystocentesis, the bacterial culture results were negative.

**TABLE 4:** Bacterial organisms isolated from 24 aerobic and anaerobic urine cultures in 21 guinea pigs with cystitis.

Isolated organism	Frequency (*n*)	Percentage (%)
*Corynebacterium renale*	12	50.0
*Staphylococcus* ^a^	5	20.8
*Aerococcus viridans* ^b^	3	12.5
*Escherichia coli*	2	8.3
Viridans group streptococci	1	4.2
Mixed flora^c^	1	4.2

^a^ *Staphylococcus* included multiple species: *Staphylococcus xylosus* (*n* = 4, one co‐isolated with *Acinetobacter lwoffii*), other *Staphylococcus* sp. (*n* = 1).

^b^ Co‐isolation with *Acinetobacter* sp. in a single microbiological urine culture (*n* = 1).

^c^ Co‐isolation of *Enterobacter* sp., *A. viridans* and *Mammaliicoccus sciuri*.

*Corynebacterium renale* was the most frequently isolated bacterium, accounting for 50% of positive cultures. Over time, the prevalence of *C. renale* as a causative agent of cystitis showed a notable increase: detected in 43% of cases before 2021 and rising to 64% in subsequent years.

Among guinea pigs with chronic cystitis and more than two recurrences or reinfections (*n* = 13), *C. renale* was detected in seven of nine available urine cultures.

In contrast, among 33 animals that underwent follow‐up urinalysis, those with favourable outcomes, defined as either no recurrence (*n* = 5) or a period without observable clinical signs exceeding 29 days (*n* = 12), showed no evidence of *C. renale* in their initial cultures. Instead, other organisms such as *Staphylococcus* spp., *Aerococcus viridans*, *Acinetobacter* spp. and viridans group streptococci were isolated. The guinea pig in which the viridans group streptococci were cultured was diagnosed with malignant lymphoma. Following a prolonged duration of clinical sign resolution, a new infection with *C. renale* was identified in five of seven guinea pigs (71.4%) that underwent a second urine culture.

Regarding housing conditions, of 21 guinea pigs for which microbiological cultures were performed, 16 were kept in groups, two with a single companion, one alone and two had unknown housing status. *C. renale* was predominantly isolated from guinea pigs housed in groups (*n* = 10), with only one case involving an animal kept with a single companion, and one from an animal with unknown housing. In one co‐housed pair, both guinea pigs had *C. renale* infections. In the other, one was infected with viridans group streptococci and the other with *Staphylococcus xylosus*.

#### Antibiotic resistance

In total, 24 urine cultures were obtained from 21 guinea pigs, and 19 antibiograms were available. An antimicrobial inhibitor test was performed in 11 cases, of which nine were negative and two were positive. Urine culture was initiated at the first presentation to the clinic in nine animals, four of which were already receiving antibiotic treatment at the time of referral. Therefore, only five animals had not received treatment at the time of specimen collection. Additionally, 15 urine cultures were conducted at later disease stages, with the longest interval between the onset of clinical signs and microbiological testing being 464 days. Trends in antimicrobial resistance over the years were analysed according to guidelines for antibiotic use in small animals.^14^

Susceptibility testing revealed the resistance patterns listed in Table 5.

**TABLE 5:** Antibiotic susceptibility profiles of cystitis pathogens in guinea pigs based on 19 susceptibility tests (2017–2022).

Bacterial species	Year	Group 1		Group 2					Group 3			
		TMS	ENR	CHL	DOX	MAR	PRAD	TUL	AZM	CLA	AMK	NIT
*Escherichia coli*	2017	R	R	S	R	R	R	R	R	R	S	S
	2021	S	S	S	S	S	S			S	S	R
*Corynebacterium renale*	2015		I	S	S	I	I	I	I	I		S
	2021	S + R	R	S	S	R	S + I + R	S	S		S	
	2022	S + R	S	S	S	S	S	S	S + I		S	
*Staphylococcus* spp.	2014	S	S	S	S		S		S	S	S	S
	2017	S	I	S	S	R	I		I	I	S	S
	2022	S	I	R	R		I	R	R	I	S	
*Aerococcus viridans*	2021	R	I	S	S	I	S	S	S		S	
	2022	R	I	S	S	I	S	S	S		S	
*A. viridans* and *Acinetobacter* sp.	2020	S	S	S		S		S	S		S	
*Acinetobacter lwoffii* and *Staphylococcus xylosus*	2021	R	R	S	S	R	R	R	R		S	

*Note*: Symbol ‘+’ denotes multiple susceptibility tests performed in a given year with varying results. Classification of authorisation status—Group 1: authorised for guinea pigs in Germany. Group 2: authorised for other animal species in Germany; use in guinea pigs requires prescription under the veterinary prescription cascade. Group 3: not authorised for veterinary use in Germany (human medicine, reserve antibiotics).

Abbreviations: AMK, amikacin; AZM, azithromycin; CHL, chloramphenicol; CLA, clarithromycin; DOX, doxycycline; ENR, enrofloxacin; I, intermediate; MAR, marbofloxacin; NIT, nitrofurantoin; PRAD, pradofloxacin; R, resistant; S, sensitive; TMS, trimethoprim‒sulphadiazine; TUL, tulathromycin.

Disregarding the temporal course of resistance, the overall sensitivity and resistance patterns of the aforementioned bacterial isolates are presented in Table 6. High levels of resistance to fluoroquinolones were observed, while most isolates were susceptible to chloramphenicol.

**TABLE 6:** Overall antimicrobial sensitivity and resistance based on 19 susceptibility tests for cystitis pathogens in guinea pigs (2017–2022).

Antimicrobial substance	Sensitivity (%)	Intermediate efficacy (%)	Resistance (%)
Chloramphenicol	94.7	0	5.3
Doxycycline	88.9	0	11.9
Azithromycin	64.8	17.6	17.6
Trimethoprim‒sulphadiazine	63.2	0	36.8
Marbofloxacin	38.9	16.7	44.4
Enrofloxacin	36.8	26.4	36.8

*Note*: Cystitis pathogens—*Escherichia coli*, *Corynebacterium renale*, *Staphylococcus* spp., *Aerococcus viridans*, *A. viridans* in combination with *Acinetobacter* sp. and *Acinetobacter lwoffii* in combination with *Staphylococcus xylosus*.

#### Radiography

Of 46 animals, 41 underwent radiological examination. Radiographically detectable urolithiasis (Figure 1) was observed in 22% (9/41) of guinea pigs; solitary uroliths were identified in 12 animals (29%). A detailed analysis of urinary bladder radiological features in a subset of 24 animals from the present population has been presented previously, showing that a bladder appearing denser, dilated and extending cranially, with a vertebral bladder score of greater than 1.3 lumbar vertebral units, is indicative of cystitis.^15^

#### Sonography

Ultrasonography was performed in 43 guinea pigs, with 13 undergoing follow‐up examinations (up to five during the disease course). The main findings included ascites, peritonitis, urinary bladder sludge, urolithiasis and pyelectasia (Table 7). In 14 animals (32.5%), the urinary bladder wall appeared irregular or thickened.

**TABLE 7:** Ultrasound findings in 43 guinea pigs with clinical signs of cystitis.

Clinical feature	Frequency (*n*)	Percentage (%)
Urinary bladder sludge	27	62.8
Peritonitis	14	32.5
Ascites	12	27.9
Urolithiasis	9	20.9
Pyelectasia	5	11.6

#### Blood analysis

Blood tests were conducted on 20 guinea pigs, of which 11 (55%) showed no abnormalities. The results are presented in Table 8. Azotaemia was observed in three animals (15%); in two cases, this was post‐renal due to urethral obstruction, while in the third case, renal azotaemia was diagnosed due to primary kidney disease. A more detailed breakdown of laboratory findings is provided in Table A1. Owing to the small number of observations per parameter, statistical analysis of blood parameter changes was only performed for monocytes (U/L) and total protein (g/dL). Although monocyte counts were above the upper reference interval in four animals, this increase was not statistically significant according to Wilcoxon signed‐rank test results (*p* = 0.125). In contrast, the total protein was significantly reduced compared with the lower reference interval (*p* = 0.042).

**TABLE 8:** Blood test findings in 20 guinea pigs with clinical signs of cystitis.

Parameter	Frequency (*n*)	Percentage (%)
Unremarkable	11	55
Azotaemia	3	15
Increased liver enzymes	3	15
Hyperbilirubinaemia	2	10
Hypoproteinaemia	5	25
Hypoglycaemia	2	10
Hypocalcaemia	2	10
Hypophosphataemia	2	10
Anaemia	2	10
Monocytosis	4	20

### Therapy

#### Analgesics

Meloxicam (0.2‒1.5 mg/kg once to twice daily, not to exceed 1.5 mg/kg/day) and metamizole (50 mg/kg three times daily) were administered for pain management. The administration regimens and durations are summarised in Table 9. Chronic cases were often managed with long‐term analgesic therapy. Notably, one guinea pig with bacteriuria caused by *C. renale* survived for 3 years without antimicrobial treatment but received daily meloxicam and metamizole.

**TABLE 9:** Analgesic administration in 46 guinea pigs with clinical signs of cystitis.

Pain management approach	Frequency (*n*)	Percentage (%)
Analgesic treatment regimen		
Meloxicam only	18	39.1
Metamizole only	1	2.2
Alternating meloxicam + metamizole	8	17.4
Simultaneous meloxicam + metamizole	19	41.3
Duration of administration (days)		
<7	4	8.7
7	8	17.4
14	4	8.7
21	2	4.3
Continuous	4	8.7
Unknown	24	52.2

#### Antimicrobial therapy

Antimicrobial treatment was administered to 45 of 46 guinea pigs (97.8%) at some point during the course of cystitis. Six antimicrobial agents were used: marbofloxacin (4 mg/kg once daily), enrofloxacin (5 mg/kg twice daily), trimethoprim‒sulphadiazine (40 mg/kg twice daily), azithromycin (15 mg/kg once daily), chloramphenicol (50 mg/kg twice daily) and metronidazole (25 mg/kg twice daily).

Of referred guinea pigs, 14 (30.4%) had already received enrofloxacin and remained on treatment at the time of referral. Antimicrobial therapy was initiated in 30 animals (65.2%) based on clinical and cytological findings at their initial presentation to our clinic. In one animal, antibiotic treatment was started only during the follow‐up examination. Fluoroquinolones were the first‐choice treatment in 34 of 45 guinea pigs (75.6%), with enrofloxacin administered to 20 guinea pigs and marbofloxacin to 14 guinea pigs. Trimethoprim‒sulphadiazine was used as the initial antimicrobial agent in 11 animals (24.4%). Treatment duration ranged from 5 days to lifelong administration, primarily via the oral route. Retrospectively, the number of animals that received an initial subcutaneous antibiotic injection could not be determined.

In three cases, antimicrobial therapy was modified based on susceptibility testing. Follow‐up urinalysis confirmed bacterial eradication in 29 animals (63.0%), whereas 17 animals (37.0%) showed persistent bacteriuria or experienced recurrence.

Six animals developed chronic cystitis, with *C. renale* detected in four cases. Additionally, *S. xylosus* was identified in one of these cases during a follow‐up examination conducted 1 year later. Five of these six chronic cases were treated continuously with trimethoprim‒sulphadiazine; however, treatment discontinuation without veterinary guidance resulted in rapid deterioration and euthanasia in three animals.

Among the 33 animals that underwent follow‐up urinalysis, two (6.1%) required combination therapy with trimethoprim–sulphadiazine and marbofloxacin due to persistent infections involving *C. renale* and *S. xylosus*. Some animals received up to six different antimicrobial agents. Treatment modifications were primarily due to persistent infection (17 cases), drug intolerance (seven cases) or delayed susceptibility testing results (three cases).

#### Glucocorticoids

Glucocorticoids were administered in three cases, not as treatment for cystitis, but for newly diagnosed conditions. Indications included suspected neoplasia in one animal, confirmed lymphoma in another, and progressive weight loss of unknown origin in the third. In all cases, glucocorticoids were initiated only after completing cystitis therapy. Based on available follow‐up data (two out of three cases), no acute deterioration in urinary findings was observed following glucocorticoid administration.

#### Plant‐based medicines

Herbal medicines were administered to 20 guinea pigs (43.5%), including 12 of 33 animals that underwent follow‐up examinations. However, due to the highly variable clinical courses, it was not possible to assess the specific effects of herbal therapy. The herbal remedies used are listed in Table A2.

#### Surgical procedures in the study population

Surgical procedures were performed in 12 of 46 guinea pigs (26%), comprising six cystotomies and six urethrotomies conducted for the removal of solitary uroliths. Among animals undergoing cystotomy, five were female and one was male, while all animals undergoing urethrotomy were female. All animals survived the procedures, with no peri‐ or postoperative arrests, and all survived to hospital discharge. One female guinea pig was euthanased 4 days post‐cystotomy (2 days after discharge) owing to deterioration of general condition.

Survival was analysed using the Kaplan–Meier method, with time to death as the response variable. Animals were censored if the exact survival time was unknown (lost to follow‐up). The median survival was 263 days (95% confidence interval: 169–357 days) for the cystotomy group and 36 days (95% confidence interval: 0–552 days) for the urethrotomy group. The log‐rank (Mantel–Cox) and Breslow (generalised Wilcoxon) tests indicated no significant difference in survival between surgical groups (*p* = 0.702 and 0.665, respectively). Owing to the small number of cases and events, multivariate Cox regression analysis was not conducted.

### Follow‐up

Of 46 guinea pigs, 84.8% underwent long‐term follow‐up, with 25 animals (54.3%) monitored until death. The median survival time was 249 days (range: 2–911 days). Additionally, 18 animals (39.1%) experienced 24 documented periods without observable clinical signs, with a median duration of 58 days (range: 11–611 days). Recurrence and reinfection were observed in 17 animals (37.0%) (Table 10). In three cases, urine culture confirmed reinfection, while classification in the remaining cases was based on timeline criteria due to the absence of follow‐up urine cultures.

**TABLE 10:** Frequencies of recurrence and reinfection in 17 guinea pigs with clinical signs of cystitis.

Type of occurrence	Frequency of occurrences (*n*)	Number of animals (*n*)	Percentage (%)
Recurrence	1	6	35.3
Reinfection	2	8	47.0
	4	2	11.9
	8	1	5.9

### Outcome

Of the total study population, 22 guinea pigs (47.8%) were euthanased, while four animals (8.7%) died acutely. The reasons for euthanasia are detailed in Table A3, with cystitis identified as the primary indication in 50% of cases. Among the guinea pigs included in the follow‐up urinalysis cohort, eight were euthanased due to cystitis. Urine cultures were available for four of these animals, all of which tested positive for *C. renale*. A review of owner compliance revealed poor adherence to treatment protocols, particularly among patients with chronic disease and multiple recurrences of *C. renale* infections. In five cases, medications were not administered continuously, and in three cases, all medications were discontinued without veterinary guidance.

## DISCUSSION

Owing to its retrospective nature, this study has certain limitations. Data collection varied among clinicians, and no standardised scale was used to grade cytological urinalysis. Moreover, cytological detection of bacteriuria in free‐catch urine is considered unreliable without a positive urine culture, as contamination from the collection method may occur. Urine cultures were not performed in all cases, and pathology results were unavailable to explore underlying pathomechanisms. Additionally, inconsistent use of ultrasound limited precise identification of bacterial‐infection sources. Secondary infections, neoplasia and accessory glands in male guinea pigs may also contribute to bacteriuria. Furthermore, assessment of antimicrobial resistance was limited, particularly in samples collected before 2017.

Only approximately one‐third of guinea pigs suspected of having cystitis underwent sufficient diagnostic evaluation to be included in the study. Financial constraints likely led owners to choose symptomatic treatment over further diagnostics.^16^, ^17^ Consistent with previous research, females were predominantly affected.^1^, ^2^, ^3^ The mean age was 4.7 ± 1.5 years, exceeding the average of 3 years reported in similar studies.^1^, ^2^

Regarding diagnostic methods, urine samples were primarily collected by manual bladder expression. While cystocentesis is recommended in dogs and cats to avoid bacterial contamination,^7^, ^10^ it is invasive and may cause complications or pain.^18^ Furthermore, although catheterisation allows targeted sample collection, it often requires sedation; carries a risk of iatrogenic injury, particularly in male guinea pigs owing to their anatomical constraints; and may inadvertently introduce bacteria into the urinary tract.^19^ Evidence from dogs demonstrates that spontaneous urine samples, when appropriately cooled and cultured on the same day using quantitative culture methods with a diagnostic threshold of 100,000 or more colony‐forming units per millilitre, provide 94% sensitivity and specificity for detecting significant bacteriuria.^20^ Furthermore, studies of the urinary microbiome in dogs^21^ and humans^22^ have successfully utilised spontaneous urine samples for bacterial analysis. Established cut‐off values for bacterial counts enable discrimination between contamination and clinically significant bacteriuria in dogs and cats.^10^ Taken together, these findings support manual bladder expression as a viable alternative in guinea pigs, balancing diagnostic reliability with the minimisation of procedural risk and animal discomfort.

With respect to bacterial agents, our findings partly align with data from dogs and cats, where the most frequently isolated bacteria in UTIs include *Escherichia coli*, *Enterococcus* spp., *Staphylococcus* spp., *Streptococcus* spp., *Enterobacter* spp., *Proteus* spp., *Klebsiella* spp., *Pseudomonas* spp., *Pasteurella* spp. and *Corynebacterium* spp.^23^, ^24^, ^25^ However, in contrast to dogs, where recurrent or chronic cystitis is typically caused by *E. coli*, *Klebsiella* spp., *Staphylococcus* spp., *Enterococcus* spp., *Proteus* spp. and *Pseudomonas* spp.,^26^, ^27^ our findings linked chronic cystitis and recurrent infections in guinea pigs predominantly to *C. renale*. This Gram‐positive, rod‐shaped bacterium attaches to urogenital epithelial cells via pili,^4^, ^28^ persists in the environment for weeks,^29^ and is challenging to eradicate.^28^ Preferring damaged tissue,^28^ it thrives in alkaline urine^30^ and is frequently isolated in guinea pigs with urolithiasis and cystitis.^4^, ^5^, ^31^ Severe infections with *C. renale* have been reported in rats,^32^, ^33^ mice^34^ and guinea pigs, where co‐infection with *Facklamia sourekii* and *Enterococcus casseliflavus* led to fatal sepsis in one case.^35^ Similar infections with *Corynebacterium* spp. have been observed in dogs, cats^36^ and immunocompromised humans,^37^ highlighting their potential for severe systemic complications. The increasing detection of *C. renale* in later disease stages herein further supports its role as a secondary invader that prefers pre‐damaged urinary mucosa.^28^ Bladder mucosal damage caused by rough urinary calculi likely promotes bacterial colonisation.^2^ Given that urolithiasis was present in nearly a quarter of cases and was the leading cause of surgery, our findings support previous research indicating a correlation between cystitis and urolithiasis in guinea pigs.^2^, ^4^, ^5^, ^31^, ^38^ As with cystitis, urolithiasis primarily affects middle‐aged guinea pigs.^39^ A recent study showed that male guinea pigs are more frequently affected by urolithiasis,^39^ in contrast to earlier findings indicating a slight female predominance.^5^ Among guinea pigs presenting with both urolithiasis and bacterial cystitis, no sex predisposition was evident.^4^ Distinct sex‐related patterns are also apparent in urolith localisation, with urethroliths occurring more frequently in females, and cystoliths being more common in males.^1^ In dogs, a correlation between uroliths and *Staphylococcus* spp. is suspected, and *Staphylococcus* spp. are often isolated in cases of recurrent or chronic infections.^27^ *Staphylococcus* spp. were the second most frequently isolated bacterial agents herein, consistent with previous research identifying these bacteria as common pathogens in guinea pigs with cystitis.^1^, ^2^, ^40^

Compared to Behrens et al.,^41^ our study demonstrates markedly better postoperative outcomes. While their study reported perioperative mortality in a proportion of guinea pigs, all animals undergoing surgery in our cohort survived the procedure and were discharged from the hospital. This difference may reflect variations in case numbers, surgical techniques, postoperative care or overall health status of the animals.

Metamizole was selected because of its analgesic and antispasmodic properties^42^ without intestinal peristalsis impairment, as demonstrated in vitro in guinea pigs.^42^, ^43^ Furthermore, metamizole has in vivo antinociceptive effects in rats via the activation of endogenous opioidergic pathways within the descending pain‐modulation system,^44^ suggesting potential systemic analgesic effects across species.

In humans, metamizole is associated with agranulocytosis development,^45^ leading to restrictions or bans in several countries. In veterinary medicine, Heinz‐body anaemia following metamizole administration has been described in multimorbid dogs.^46^ However, to the authors’ knowledge, agranulocytosis associated with metamizole use has not been reported in small mammals to date.^47^, ^48^ Therefore, the potential risk of rare adverse effects must be carefully weighed against expected analgesic benefits in these species.^48^

No specific monitoring protocol for adverse effects was implemented during metamizole administration herein. However, 11 guinea pigs receiving metamizole were among the animals in which blood examinations were performed during the cystitis course. Of these, two animals exhibited haematological changes consistent with anaemia. Owing to the study's retrospective nature, no causal relationship between metamizole administration and these findings could be established. Prospective studies are required to further investigate the safety profile of metamizole and potential adverse effects in guinea pigs.

In dogs and cats, group‐housed animals have increased transmission of multidrug‐resistant bacterial infections.^49^ In our guinea pig population, *C. renale* infections were also observed in animals maintained in group housing. However, the dataset is limited, and the exact mode of transmission and causality in guinea pigs remain unclear. While a potential trend may exist, further studies with larger and more balanced cohorts are required to confirm an association.

Compared to dogs, guinea pigs appear considerably more prone to reinfections and recurrences. In canine populations, only 4.5% of UTIs are recurrent or chronic.^26^ In dogs and cats, recurrence refers to a new infection caused by the same pathogen within days to weeks of completing therapy,^10^ typically indicating treatment failure. This failure is often linked to inappropriate antimicrobial selection or administration during the initial infection, potentially facilitating the emergence of resistant bacterial strains.^11^ Reinfections are caused by different pathogens and may occur at any time after the initial episode.^10^ However, distinguishing between reinfections and recurrences is often challenging, as follow‐up urine cultures are generally performed only when clinical signs reappear, rather than routinely post‐treatment. Introducing standardised post‐treatment urine‐culture protocols would enhance understanding of infection dynamics in guinea pigs. Nonetheless, owner compliance poses a persistent challenge; repeat testing rates often decline if the animal appears clinically recovered, limiting accurate monitoring and early intervention.

Finally, the unsatisfactory outcomes observed despite treatment guided by antibiotic susceptibility testing raise questions about the underlying cause of this phenomenon. Recent advances in molecular diagnostic methods, such as metagenomic next‐generation sequencing, have indicated that conventional culture may underestimate the diversity of urinary tract microbiota and potentially overlook fastidious or anaerobic organisms.^50^ Although standard culture remains the most widely available diagnostic method in veterinary practice, future studies employing metagenomic next‐generation sequencing could provide deeper insights into the urinary microbiome of guinea pigs and clarify the role of previously undetected bacteria in chronic or recurrent cystitis. Another potential explanation is that dosages and dosing intervals of prescribed antimicrobials may be insufficient to achieve effective bladder concentrations.^7^ Conversely, bacteria may not be the primary aetiological agent in guinea pigs. Similar to feline idiopathic cystitis, where subclinical bacteriuria, impaired host defences, and underlying comorbidities play important roles,^12^ an idiopathic form of UTI may exist in guinea pigs. Moreover, guinea pigs appear more prone to complicated UTIs. Unlike uncomplicated UTIs, which generally resolve with appropriate antimicrobial therapy, complicated infections, defined in dogs and cats as those associated with anatomical abnormalities, immunosuppression, frequent recurrences or persistent treatment failure,^11^, ^12^ are more difficult to manage and could explain the high rates of recurrence and therapeutic resistance observed herein.

## AUTHOR CONTRIBUTIONS

Anika Mische and Kerstin Müller contributed to the conceptualisation and design of the study. Anika Mische was responsible for the acquisition of data, and both authors contributed to the analysis and interpretation. Anika Mische drafted the manuscript, and Kerstin Müller reviewed it for important intellectual content. All authors meet the authorship criteria outlined by the journal, approved the final version of the manuscript and agree to be accountable for all aspects of the work, ensuring that any questions related to the accuracy or integrity of any part of the work are appropriately investigated and resolved.

## CONFLICT OF INTEREST STATEMENT

The authors declare no conflicts of interest that could influence or bias the content of this article.

## FUNDING INFORMATION

The authors received no specific funding for this work.

## ETHICS STATEMENT

Ethical approval was not required, as the study involved a retrospective analysis of previously collected clinical data. Owner consent was obtained for the use of the data for research purposes.

## Supporting information



Supporting Information

Supporting Information

## Data Availability

The data that support the findings of this study are available from the corresponding author upon reasonable request.
