# Supplementary material for: Cystitis in guinea pigs (Cavia porcellus): Clinical findings and treatment outcomes
Source: Vet Rec. 2026 Feb 11;199(1):e30–8. doi: 10.1002/vetr.70391 (PMC13330707; doi:10.1002/vetr.70391)
Supplement: Supplementary file 1 — Supporting Information [file VETR-199--s001.docx]

Appendix

Appendix TABLE A1. Extended haematological-parameter and biochemical-parameter deviations in guinea pigs with clinical signs of cystitis (n = 9)

| Pat | Hct (U/L) | Ref Hct | Segs (%) | Ref Segs | Lym (%) | Ref Lym | Mono (U/L) | Ref Mono | Urea (mg/  dl) | Ref Urea | Cr (**µmol/L)** | Ref Cr | TP (g/dl) | Ref TP | Alb (g/L) | Ref Alb | AST (U/L) | Ref AST | GLDH (U/L) | Ref GLDH | Bili (**µmol/L)** | Ref Bili | Phos (mmol/L) | Ref Phos | Glu (mmol/L) | Ref Glu | Ca (mmol/L) | Ref Ca |
| --- | --- | --- | --- | --- | --- | --- | --- | --- | --- | --- | --- | --- | --- | --- | --- | --- | --- | --- | --- | --- | --- | --- | --- | --- | --- | --- | --- | --- |
| 1 |  |  |  |  |  |  | 490 ↑ | <300 |  |  |  |  | 5 ↓ | 5.4-6.7 |  |  |  |  |  |  |  |  |  |  |  |  |  |  |
| 2 | 26.8 ↓ | 34.9-52.9 | 83 ↑ | 13.7-55.8 | 6 ↓ | 41.4-77.7 | 901 ↑ | <300 | 28.8 ↑ | 8.7--26 |  |  | 4.5 ↓ | 5.4-6.7 |  |  | 166 ↑ | <115 |  |  |  |  | 0.6 ↓ | 0.8-1.7 |  |  |  |  |
| 3 |  |  |  |  |  |  | 518 ↑ | <300 |  |  |  |  | 4.3 ↓ | 5.4-6.7 |  |  |  |  |  |  |  |  |  |  | 6.3 ↓ | 6.4-13.6 | 2.2 ↓ | 2.4-3 |
| 4 |  |  |  |  |  |  |  |  |  |  |  |  |  |  | 24.6 ↓ | 26-41 |  |  |  |  | <1.71 ↑ | <1.59 | 0.57 ↓ | 1.03-6.98 |  |  |  |  |
| 5 | 30.5 ↓ | 34.9-52.9 |  |  |  |  |  |  | 28 ↑ | 8.7--26 |  |  | 4.5 ↓ | 5.4-6.7 |  |  | 195 ↑ | <115 | 65 ↑ | <20 |  |  |  |  |  |  |  |  |
| 6 |  |  |  |  |  |  |  |  |  |  |  |  |  |  |  |  |  |  |  |  | <1.71 ↑ | <1.59 |  |  | 4.61 ↓ | 4.95-15.95 |  |  |
| 7 |  |  |  |  |  |  |  |  |  |  | 409 ↑ | <166 |  |  |  |  |  |  |  |  |  |  |  |  |  |  |  |  |
| 8 |  |  |  |  |  |  |  |  |  |  | 345 ↑ | <166 |  |  |  |  |  |  |  |  |  |  |  |  |  |  |  |  |
| 9 |  |  | 69 ↑ | 13.7-55.8 | 23 ↓ | 41.4-77.7 | 730 ↑ | <300 |  |  | 79.2 ↑ | 61.6 | 5.2 ↓ | 5.4-6.7 |  |  | 773 ↑ | <115 | 352 ↑ | <20 |  |  |  |  |  |  |  |  |

**Abbreviations:** Pat – Patient; Hct – Haematocrit; Segs – Segmented Neutrophils; Lym – Lymphocytes; Mono – Monocytes, Urea – Urea; Cr – Creatinine; TP – Total Protein; Alb – Albumin; AST – Aspartate Aminotransferase; GLDH – Glutamate Dehydrogenase; Bili – Bilirubin (Total); Phos – Phosphate; Glu – Glucose; Ca – Calcium (Total);

Appendix TABLE A2. Use of plant-based therapeutics in 20 guinea pigs with clinical signs of cystitis

| Preparations | Frequency  (n) | Percentage  (%) |
| --- | --- | --- |
| Rodicare uro®^1^ | 13 | 65 |
| Eurologist®^2^ | 6 | 30 |
| allrodin® UTI Kn^3^ | 2 | 10 |
| ANGOCIN®^4^ | 1 | 5 |
| UROplex®^5^ | 1 | 5 |
| Bladder and kidney tea^6^ | 5 | 25 |
| Cranberry juice | 3 | 15 |

^1^ alfavet, Neumünster, Germany

^2^ Animalherbs E.V., Coevorden, Netherlands

^3^ almapharm, Wildpoldsried, Germany

^4^ Repha, Langenhagen, Germany

^5^ bunnyNature, Melle, Germany

^6^ Bladder and kidney tea: over-the-counter herbal mixtures from drugstores (typically containing *Urtica dioica*, *Equisetum arvense*, *Solidago virgaurea*).

Appendix TABLE A3. Reasons for euthanasia in 22 guinea pigs with clinical signs of cystitis

| Category | Specific Condition | Frequency  (n) | Percentage  (%) |
| --- | --- | --- | --- |
| Cystitis-associated | Cystitis | 11 | 50 |
| Non-cystitis-associated | Dyspnoea | 2 | 9.1 |
|  | Thoracic effusion | 1 | 4.54 |
|  | Intestinal haemorrhage | 1 | 4.54 |
|  | Gastric tympany | 1 | 4.54 |
|  | Gastric torsion | 1 | 4.54 |
|  | Lymphoma | 1 | 4.54 |
|  | Osteoarthritis | 1 | 4.54 |
|  | Urethral calculus with nephropathy | 1 | 4.54 |
|  | Renal failure | 1 | 4.54 |
|  | Dental disease | 1 | 4.54 |
