# Supplementary material for: Cystitis in guinea pigs (Cavia porcellus): Clinical findings and treatment outcomes
Source: Vet Rec. 2026 Feb 11;199(1):e30–8. doi: 10.1002/vetr.70391 (PMC13330707; doi:10.1002/vetr.70391)
Supplement: Supplementary file 2 — Supporting Information [file VETR-199--s002.pdf]

Appendix

Appendix TABLE A1. Extended haematological-parameter and biochemical-parameter deviations in guinea pigs with clinical signs of cystitis (n = 9)

Pat	Hct (U/L)	Ref Hct	Segs (%)	Ref Segs	Lym (%)	Ref Lym	Mono (U/L)	Ref Mono	Urea (mg/dl)	Ref Urea	Cr (µmol/L)	Ref Cr	TP (g/dl)	Ref TP	Alb (g/L)	Ref Alb	AST (U/L)	Ref AST	GLDH (U/L)	Ref GLDH	Bili (µmol/L)	Ref Bili	Phos (mmol/L)	Ref Phos	Glu (mmol/L)	Ref Glu	Ca (mmol/L)	Ref Ca
1							490 ↑	<300					5 ↓	5.4-6.7														
2	26.8 ↓	34.9-52.9	83 ↑	13.7-55.8	6 ↓	41.4-77.7	901 ↑	<300	28.8 ↑	8.7--26			4.5 ↓	5.4-6.7			166 ↑	<115					0.6 ↓	0.8-1.7				
3							518 ↑	<300					4.3 ↓	5.4-6.7											6.3 ↓	6.4-13.6	2.2 ↓	2.4-3
4															24.6 ↓	26-41					<1.71 ↑	<1.59	0.57 ↓	1.03-6.98				
5	30.5 ↓	34.9-52.9							28 ↑	8.7--26			4.5 ↓	5.4-6.7			195 ↑	<115	65 ↑	<20								
6																					<1.71 ↑	<1.59			4.61 ↓	4.95-15.95		
7											409 ↑	<166																
8											345 ↑	<166																
9			69 ↑	13.7-55.8	23 ↓	41.4-77.7	730 ↑	<300			79.2 ↑	61.6	5.2 ↓	5.4-6.7			773 ↑	<115	352 ↑	<20								

Abbreviations: Pat – Patient; Hct – Haematocrit; Segs – Segmented Neutrophils; Lym – Lymphocytes; Mono – Monocytes, Urea – Urea; Cr – Creatinine; TP – Total Protein; Alb – Albumin; AST – Aspartate Aminotransferase; GLDH – Glutamate Dehydrogenase; Bili – Bilirubin (Total); Phos – Phosphate; Glu – Glucose; Ca – Calcium (Total);

Appendix TABLE A2. Use of plant-based therapeutics in 20 guinea pigs with clinical signs of cystitis

Preparations	Frequency (n)	Percentage (%)
Rodicare uro®[1]	13	65
Eurologist®[2]	6	30
allrodin® UTI Kn[3]	2	10
ANGOCIN®[4]	1	5
UROplex®[5]	1	5
Bladder and kidney tea[6]	5	25
Cranberry juice	3	15

[1] alfavet, Neumünster, Germany

[2] Animalherbs E.V., Coevorden, Netherlands

[3] almapharm, Wildpoldsried, Germany

[4] Repha, Langenhagen, Germany

[5] bunnyNature, Melle, Germany

[6] Bladder and kidney tea: over-the-counter herbal mixtures from drugstores (typically containing *Urtica dioica*, *Equisetum arvense*, *Solidago virgaurea*).

Appendix TABLE A3. Reasons for euthanasia in 22 guinea pigs with clinical signs of cystitis

Category	Specific Condition	Frequency (n)	Percentage (%)
Cystitis-associated	Cystitis	11	50
Non-cystitis-associated	Dyspnoea	2	9.1
	Thoracic effusion	1	4.54
	Intestinal haemorrhage	1	4.54
	Gastric tympany	1	4.54
	Gastric torsion	1	4.54
	Lymphoma	1	4.54
	Osteoarthritis	1	4.54
	Urethral calculus with nephropathy	1	4.54
	Renal failure	1	4.54
	Dental disease	1	4.54
